# Mitochondrial *Glrx2* Knockout Augments Acetaminophen-Induced Hepatotoxicity in Mice

**DOI:** 10.3390/antiox11091643

**Published:** 2022-08-24

**Authors:** Jing Li, Xuewen Tang, Xing Wen, Xiaoyuan Ren, Huihui Zhang, Yatao Du, Jun Lu

**Affiliations:** 1 Key Laboratory of Luminescence Analysis and Molecular Sensing, Ministry of Education (Southwest University), College of Pharmaceutical Sciences, Southwest University, Chongqing 400715, China; 2 West China School of Public Health and West China Fourth Hospital, Sichuan University, Chengdu 610041, China; 3 Division of Biochemistry, Department of Medical Biochemistry and Biophysics, Karolinska Institutet, SE-171 77 Stockholm, Sweden; 4 Shanghai Institute of Immunology, Shanghai Jiao Tong University School of Medicine, Shanghai 200025, China; 5 Faculty of Basic Medicine, Shanghai Jiao Tong University School of Medicine, Shanghai 200025, China; 6 Ministry of Education-Shanghai Key Laboratory of Children’s Environmental Health, Institute of Early Life Health, Xinhua Hospital, Shanghai Jiao Tong University School of Medicine, Shanghai 200092, China

**Keywords:** glutaredoxin2, acetaminophen, hepatotoxicity, thioredoxin, glutaredoxin system, glutathionylation, redox regulation

## Abstract

Acetaminophen (APAP) is one of the most widely used drugs with antipyretic and analgesic effects, and thus hepatotoxicity from the overdose of APAP becomes one of the most common forms of drug-induced liver injury. The reaction towards thiol molecules, such as GSH by APAP metabolite, *N*-acetyl-*p*-benzo-quinonimine (NAPQI), is the main cause of APAP-induced hepatotoxicity. However, the role of many other thiol-related regulators in toxicity caused by APAP is still unclear. Here we have found that knockout of the *Glrx2* gene, which encodes mitochondrial glutaredoxin2 (Grx2), sensitized mice to APAP-caused hepatotoxicity. *Glrx2* deletion hindered Nrf2-mediated compensatory recovery of thiol-dependent redox systems after acetaminophen challenge, resulting in a more oxidized cellular state with a further decrease in GSH level, thioredoxin reductase activity, and GSH/GSSG ratio. The weakened feedback regulation capacity of the liver led to higher levels of protein glutathionylation and thioredoxin (both Trx1 and Trx2) oxidation in *Glrx2^−/−^* mice. Following the cellular environment oxidation, nuclear translocation of apoptosis-inducing factor (AIF) was elevated in the liver of *Glrx2^−/−^* mice. Taken together, these results demonstrated that mitochondrial Grx2 deficiency deteriorated APAP-induced hepatotoxicity by interrupting thiol-redox compensatory response, enhancing the AIF pathway-mediated oxidative damage.

## 1. Introduction

As a common drug-caused adverse effect, liver injury can elicit liver failure, or even death [1], which is a great challenge for the drug’s application. This gives rise to the need to uncover the molecular mechanism of drug-induced liver injury. Accumulating studies reported that oxidative stress could be a primary cause of liver damage induced by drugs [2,3,4], such as acetaminophen [5]. Acetaminophen (APAP) is generally used for antipyretic and analgesic effects worldwide. However, its hepatotoxicity caused by intentional or unintentional overdose usage largely threatens people’s health and even life [6,7]. Therefore, it is of great urgency to understand the underlying mechanism of APAP poisoning. Although various cellular events are involved in this hepatotoxicity process, mitochondrial dysfunction and oxidative stress have currently been identified as the predominant causes of APAP-induced liver damage [8,9,10]. Mechanistically, the APAP metabolite, *N*-acetyl-*p*-benzo-quinonimine (NAPQI), depletes glutathione (GSH), inhibits thioredoxin reductases (TrxR), and modifies mitochondrial proteins, leading to inhibition of mitochondrial respiration and oxidative stress. Subsequently, apoptosis signal-regulating kinase 1 (ASK1) is activated by phosphorylating c-Jun N-terminal kinase (JNK), and p-JNK is translocated to mitochondria and induces mitochondrial permeability transition (MPT), triggering nuclear translocation of apoptosis-inducing factor (AIF) [11,12]. Indeed, the concept that GSH involves hepatotoxicity mechanisms has been well accepted, but the roles of other thiol-redox mediators, including some GSH-related enzymes such as glutaredoxins (Grx), are not well understood.

Human mitochondrial glutaredoxin 2 (Grx2, encoded by *Glrx2*), an important component in the GSH-Grx system, is a member of the Trx superfamily with a Trx-fold in its structure [13,14], which may participate in catalyzing protein glutathionylation/deglutathionylation and iron metabolism. Glutathione, an upstream electron donor that regulates Grx2 activity, has two forms: reduced GSH and oxidized GSSG. The cytosolic GSH/GSSG ratio is very high (>99%) under physiological conditions. When exposed to oxidative stress, a large number of GSHs are oxidized to GSSG, resulting in a decreased GSH/GSSG ratio. The lowered ratio of GSH/GSSG enhances the formation of protein glutathionylation [15,16] and thus affects the biological functions of glutathionylated proteins, such as mitochondrial complex 1 [17]. Very interestingly, Grx2 is present as the form of the inactivated dimer under normal conditions, with its cysteine residues in two Grx2 molecules connected through the [2Fe-2S] cluster [18]. Oxidative stress stimulates Grx2 depolymerization to produce the activated Grx2 monomer to exert its function by monothiol or dithiol mechanisms [15,19]. The deficiency of Grx2 may block the biosynthesis of the Fe-S cluster and then affect iron metabolism. This indicates the participation of Grx2 in the initiation and development of iron metabolism-related diseases, such as Parkinson’s disease [20] and cancer [21]. In addition, recent studies reported that *Glrx2* ablation enhanced high-fat diet (HFD)-induced oxidative stress, inflammatory response, insulin resistance, and mitochondrial dysfunction in the brain [22], but counteracted HFD-caused oxidative stress and weight gain in skeletal muscle [23]. Accumulating evidence manifested that *Glrx2* deficiency increased cellular sensitivity to oxidative stress [24,25,26]. Though it is known that Grx2 is a special player in defense against oxidative stress, the involvement of Grx2 in drug detoxification is rarely reported.

Oxidative stress is a major mechanism for the overdose of APAP to cause liver damage [8,27,28]. Our previous results also showed that APAP exposure resulted in a quick inhibition of TrxR and a decrease in GSH level, following a dose-dependent compensatory response for the thiol-dependent redox systems to fight excessive ROS. Whether the cellular compensatory response achieved the recovery of redox balance was indicated by the protein glutathionylation levels in the liver [29]. Considering that Grx2 is a main player in regulating the protein glutathionylation in the mitochondria, and that APAP-caused liver injury is related to dysfunction of mitochondria, we, therefore, performed the study to investigate whether Grx2 is involved in APAP detoxification. Particularly, we investigated the effects of Grx2 deficiency on the redox compensatory response caused by APAP-induced oxidative stress.

## 2. Materials and Methods

### 2.1. Reagents

APAP was obtained from Adamas-beta (Shanghai, China). Reagents used for TrxR activity determination: 5,5’-Dithiobis-(2-nitrobenzoic acid) (DTNB) and nicotinamide adenine dinucleotide phosphate (NADPH) were from Biosharp (Anhui, China); Aurothioglucose (ATG) was purchased from Wako (Osaka, Japan). Glutathione reductase (GR) and glutathione (GSH) were purchased from Solarbio (Beijing, China). *N*-ethylmaleimide (NEM) was purchased from Sigma-Aldrich (Saint Louise, MO, USA). Anti-GSH antibody (from mice) was purchased from Abcam (Cambridge, UK), anti-thioredoxin1 (Trx1), and anti-Trx2 antibodies were from IMCO (Stockholm, Sweden); all other antibodies used in the study were obtained from Proteintech (Hubei, China). Sep-Pak C18 cartridge was purchased from Waters Corporation (Milford, MA, USA).

### 2.2. Mice construction and Genotyping

Wild-type (WT) C57BL/6J male mice were purchased from Hunan SJA laboratory animal company (Hunan, China). C57BL/6J *Glrx2*-deficient (*Glrx2^−/−^*) mice were obtained from the Central Institute for Experimental Animals (CIEA; http://www.ciea.or.jp, accessed on 10 January 2020), Kawasaki, Japan. All experiments were approved by the Institutional Animal Care and Use Committee (IACUC) of Southwest University with IACUC number IACUC-20190912-01.

The *Glrx2^−/−^* mice were constructed using the homologous recombination method. Specifically, upstream and downstream homologous sequences of interested *Glrx2* locus were amplified and inserted into each side of neomycin-resistance gene in targeting vector. Resultant construct was injected into C57BL/6J embryonic stem cells and crossed with C57BL/6J WT mice. Homozygous mice were screened and used to reproduce offspring.

To make sure the *Glrx2^−/−^* mice used in our experiment were indeed *Glrx2*-deficient mice, genotyping was carried out following the protocol of one-step mouse genotyping kit (Vazyme, Nanjing, CN). Then, 1–3 mm mice tail was collected and soaked in lysis buffer containing proteinase K for 20 min incubation at 55 °C to extract DNA. After centrifugation, the template DNA in supernatant was amplified using PCR with certain primers (for wild-type mice, forward: 5′-cacgaggagcacctactgtgt-3′, reverse: 5′-cctgagggagacaaagatgc-3′; for *Glrx2^−/−^* mice, forward: 5′-gcttggctggacgtaaactc-3′, reverse: 5′-cctgagggagacaaagatgc-3′) and separated in 2% agarose gel. The gel images were captured using a Gel Imager System (Sagecreation, Beijing, China).

The picture showed that the DNA molecular weight of WT and *Glrx2^−/−^* mice were in the range of 300–400 bp and 400–500 bp, respectively (Figure S1A). This could be explained by the construction procedure of *Glrx2^−/−^* mice. A neomycin-resistance gene with upstream and downstream homologous arms of *Glrx2* on each side was used to inactivate *Glrx2*; thus, using forward primers targeting neomycin-resistance gene (in *Glrx2^−/−^* mice) or *Glrx2* (in WT mice), we could obtain PCR products with different molecular weights. Further, the Grx2 protein level in mice liver was detected by Western blot. As shown, the Grx2 protein expression was not detected in *Glrx2^−/−^* mice (Figure S1B).

### 2.3. Animal Treatment

WT and *Glrx2^−/−^* male C57 BL/6J mice were housed in same conditions with a 12 h light–12 h dark cycle. After genotyping, the mice were subjected to various procedures according to different purposes. To explore the acute toxicity of APAP in WT and *Glrx2^−/−^* mice, ten WT or ten *Glrx2^−/−^* male mice were exposed to 600 mg·kg^−1^ APAP after overnight fasting, then the number and time of mice death were recorded to plot the survival curve. To understand the underlying mechanism, genotyped mice (10 male) were divided into two subgroups (5 mice each subgroup) and treated with saline and 300 mg·kg^−^^1^ APAP, respectively, by intragastric injection. At 1 h and 6 h post-APAP administration, mice were sacrificed. Blood and liver tissues were collected for further analysis.

### 2.4. Liver Injury Evaluation

Liver damage was evaluated using serum biomarkers, including aspartate aminotransferase (AST), alanine aminotransferase (ALT), and hematoxylin-eosin (HE) staining. Collected blood was kept still at room temperature for 1 to 2 h, then centrifuged at 2500 g for 20 min after clotting. The supernatant was collected and processed by AST and ALT activity analysis by automatic biochemistry analyzer. A small piece of fresh liver tissue was cut and kept in 4% (*w*/*v*) paraformaldehyde for HE staining and nuclear translocation detection of AIF. Three mice in each group were used to analyze, and the representative one was shown.

### 2.5. Total Glutathione Assay

Total glutathione content was detected using a 5,5’-Dithiobis-(2-nitrobenzoic acid) (DTNB) assay in a 96-well plate [29]. In brief, 5 μg liver protein was incubated with detection working solution containing 100 μM nicotinamide adenine dinucleotide phosphate (NADPH) and 50 nM glutathione reductase (GR) in Tris-HCl, EDTA (TE) buffer, pH 7.4 for 5 min at room temperature in sample wells, with only TE buffer containing 100 μM NADPH in reference wells. After that, the mixture of 2 mM DTNB and 100 μM NADPH was added to start the reaction, and the absorbance value at 412 nm was immediately monitored by microplate reader (Biotek, Winooski, VT, USA) for 5 min with 1 min-interval. The slope was calculated to represent the GSH level. Relative GSH level in the sample was calculated by subtracting the slope of the reference well from that of the sample well. The data were normalized to the value obtained from the WT group treated with saline. The liver extracts from all five mice in each group were subjected to analysis, and the data in each mouse was measured in triplicate.

### 2.6. TrxR Activity Assay

TrxR activity was indirectly measured by a modified DTNB assay [30]. Briefly, 25 μg protein was incubated with 100 μM NADPH in TE buffer, pH 7.4 for 2 min at room temperature, then 300 nM ATG, a specific TrxR inhibitor, was added in reference wells. After 5 min incubation, 100 μL solution containing 100 μM NADPH and 2 mM DTNB was added to start the reduction reaction. The absorbance change was monitored with the same procedure as used for total glutathione detection. The slope was used to represent the enzyme activity.

### 2.7. Protein Expression Analysis

After treatment with 300 mg·kg^−1^ APAP for the indicated time, the mice were sacrificed, and liver tissues were collected. About 0.5 g liver tissue was cut into small pieces and rinsed with cold saline twice, then lysed in 1 mL RIPA (containing 1 mM PMSF) using an electric homogenizer. Lysates were separated by centrifugation at 13,000 rpm, and protein concentration of obtained supernatant was detected by BCA assay. Equal amounts of proteins were subjected to SDS-PAGE for separation and further Western blotting analysis. The dilution times for primary antibodies were 1:3000 (HO-1), 1:1000 (Trx1/2, TrxR1/2), and 1:500 (Grx2). GAPDH (1:5000) was used as the loading control.

### 2.8. Protein Redox Status Detection

Protein thiol modifications, including glutathionylation and protein-Trx interaction, were detected based on the alkylation of iodoacetamide (IAM) on free thiols [29,30]. Fresh liver tissue (about 0.1 g) was cut into small pieces and washed with pre-cold saline twice to clear blood. Then, the washed liver tissue pieces were added to the RIPA lysis buffer containing 1 mM phenylmethylsulfonyl fluoride (PMSF) and 10 mM IAM, and homogenized with an electric homogenizer (Servicebio, Wuhan, China) at 60 Hz for 2 min. Resultant lysate was centrifugated at 13,000× *g* for 10 min to collect proteins in supernatant. After protein concentration measurement, the proteins (25 μg) were separated with non-reducing SDS-PAGE and transferred to a polyvinylidene fluoride (PVDF) membrane. Glutathionylated proteins and Trxs were detected with corresponding antibodies at different dilutions (anti-GSH: 1:5000 dilution; anti-Trx1/2:1:1000 dilution). Finally, the proteins in the membrane were captured by chemiluminescence imaging system (Clinx, Shanghai, China). For glutathionylation detection, Coomassie brilliant blue staining gel with equal content of proteins was used as loading control. Five mice in each group were used to analyze, and representative one was shown.

### 2.9. GSH/GSSG Assay

GSH/GSSG ratio was measured using the method described by Adams J.D. et al. [31] and Motterlini R. et al. [32] with some minor modifications. Briefly, after washing with cold saline, about 10 mg fresh liver tissue was homogenized in 1 mL buffer 10 mM *N*-ethylmaleimide (NEM) dissolved in buffer 1 (containing 100 mM PBS and 5 mM EDTA, pH 6.5) for GSSG detection, and another 10 mg fresh liver tissue was homogenized in 1 mL, 10 mM DTNB dissolved in buffer 2 (containing 100 mM PBS and 5 mM EDTA, pH 7.5) for total glutathione detection. Then, both were processed for centrifugation at 4 °C, 12,000× *g* for 10 min. Supernatant was collected, and its protein concentration was determined by Bradford assay and calculated using bovine serum albumin (BSA) as standard. In GSSG detection assay, excessive free NEM in samples was removed using the C18 Sep-Pak cartridge. Then, 50 μL eluate was added to 96-well plates and incubated with 50 μL mixture containing 0.1 U GR and 50 nmol DTNB in buffer 1 for 1 min. Subsequently, 44 nmol NADPH in 100 μL buffer 1 was added to start the reaction. By monitoring the absorbance for 5 min with 1 min-interval using microplate reader, the slope (absorbance change/min) was obtained, and the GSSG content was calculated using a standard curve. Same to the GSSG detection, after 50-fold dilution, the sample was subjected to total glutathione measurement using the method described above. Each sample was detected in triplicate. Reduced GSH was calculated according to the formula: Reduced GSH = total glutathione − GSSG × 2.

### 2.10. AIF Nuclear Translocation Detection

Mice were sacrificed at the indicated time point, and a small piece of fresh liver tissue was kept in 4% paraformaldehyde for apoptosis-inducing factor (AIF) nuclear translocation detection using AIF antibody (Proteintech, Wuhan, China). The representative image was captured by fluorescence microscopy (NIKON Eclipse Ci, Tokyo, Japan).

### 2.11. MDA Level Detection

Malondialdehyde (MDA) level in the liver was measured using a lipid peroxidation MDA assay kit (Beyotime, Shanghai, China) following the procedure described in the protocol. Fresh liver tissue was cut into small pieces and washed with cold saline to remove blood, then homogenized with PBS buffer (containing 1 mM PMSF). After centrifugation at 13,000× *g* for 10 min, the precipitate was discarded, and protein concentration of the supernatant was measured by BCA assay. MDA in the sample reacted with thiobarbituric acid (TBA) to produce a red MDA-TBA adduct which had the maximum absorption at 535 nm. By detecting the absorbance at 535 nm, the MDA level was quantified using a MDA standard curve. Four mice in each group were included to analyze.

### 2.12. Statistical Analysis

All data are shown as mean ± SEM. Western blot results were quantified by Image J. Statistical difference was assessed by GraphPad Prism using two-way ANOVA. A value of *p* < 0.05 was indicated to be significant.

## 3. Results

### 3.1. Glrx2 Deficiency Exacerbated APAP Caused Acute Hepatotoxicity

**Figure 1:**
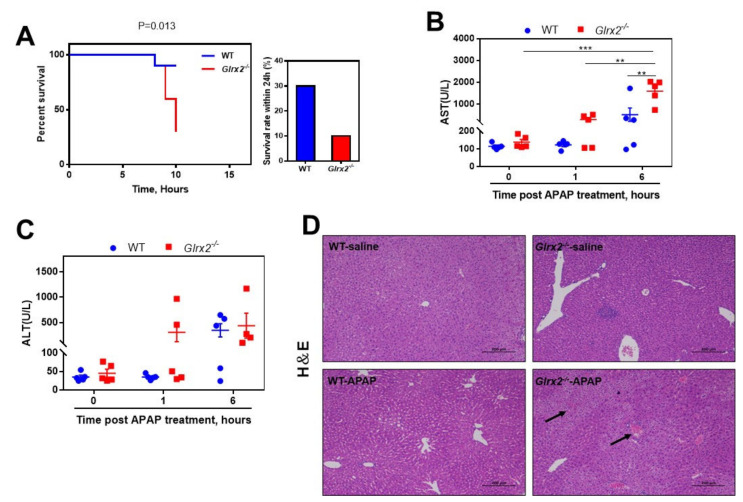
*Glrx2* knockout increased liver sensitivity to acetaminophen (APAP) challenge. (**A**) Survival curve and survival rate of mice exposed to APAP challenge. Both WT and *Glrx2^−/−^* mice were treated with 600 mg·kg^−1^ APAP, and the survival of mice were monitored to obtain the survival curve within 10 h and survival rate within 24 h, *n* = 10. (**B**,**C**) The activities of aspartate aminotransferase (AST) and alanine aminotransferase (ALT) in serum in mice after treatment with 300 mg·kg^−1^ APAP. The statistical difference was analyzed by two-way ANOVA. Data are shown as mean ± SEM, *n* = 5, ** *p* < 0.01; *** *p* < 0.001. (**D**) Pathological morphology of liver from mice treated with 300 mg·kg^−1^ APAP at 6 h after administration, assessed by hematoxylin-eosin (HE) staining. Arrows point to necrotic hepatocytes.

To see the effect of Grx2 on the APAP-caused toxicity, both WT and *Glrx2^−/−^* mice were exposed to a lethal dose of 600 mg·kg^−1^ APAP, and the death time and number of mice were monitored to obtain a Kaplan–Meier survival curve and survival percentage. At 8 h post-APAP administration, in both WT and *Glrx2^−/−^* groups, one mouse died. No mouse died in the WT group at a subsequent 2 h, while another three mice died at 9 h and 10 h in the *Glrx2^−/−^* group, respectively. After 24 h, 3 mice survived in the APAP-treated WT group and 1 in the APAP-treated *Glrx2^−/−^* group. This result showed that there was a significant difference in APAP toxicity in WT and *Glrx2^−/−^* mice (*p*-value: 0.013), and *Glrx2^−/−^* mice seem to be more sensitive than WT mice to the APAP challenge (Figure 1A).

To further explore the mechanism causing this difference, the APAP dose was decreased to 300 mg·kg^−1^ to ensure enough sample for analysis, and liver damage was assessed by transaminase activity in serum and hematoxylin-eosin (HE) staining of liver tissue. Even at 1 h post-APAP treatment, two liver damage markers, alanine transaminase (ALT) and aspartate transaminase (AST) activity, were found to be increased, indicating liver injury in two-fifths of *Glrx2^−/−^* mice, but not in WT mice (Figure 1B,C). AST and ALT increased in both genotypes of mice at 6 h after administration. Although ALT showed no difference, AST activity in *Glrx2^−/−^* mice was much higher than that in WT mice and that in *Glrx2^−/−^* mice exposed to APAP for 1 h (Figure 1B,C). HE staining of livers from the mice treated with APAP for 6 h was consistent with the above results (Figure 1D). An ordered arrangement of hepatocytes and integral cell morphology was observed in both WT and *Glrx2^−/−^* mice treated with saline, while severer liver damage characterized by a mass of hepatocytes necrosis was found in APAP-treated *Glrx2^−/−^* mice than the case in APAP-treated WT mice. These results revealed that *Glrx2* depletion exacerbated the APAP hepatotoxicity.

### 3.2. APAP Treatment Decreased Total Glutathione Content and TrxR Activity and Triggered Nrf2 Activation

**Figure 2:**
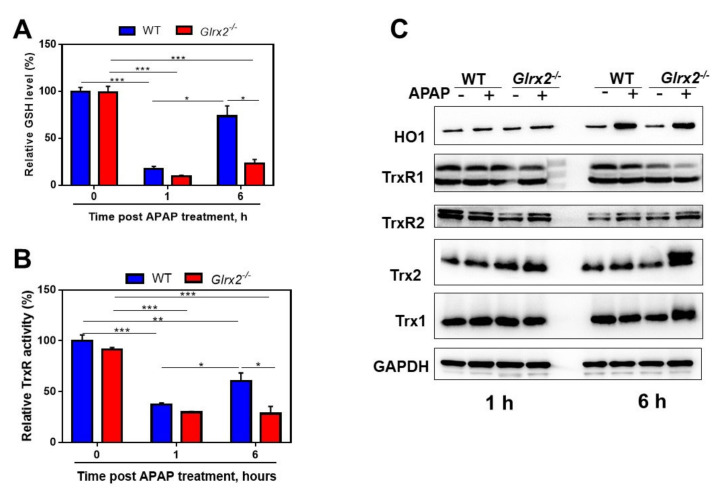
*Glrx2* deficiency weakened the compensatory response of thiol-antioxidant systems. (**A**) The effect of *Glrx2* knockout on glutathione (GSH) level in the livers from APAP-treated mice. (**B**) The effect of *Glrx2* knockout on thioredoxin reductase (TrxR) activity in livers from the mice exposed to APAP. (**A**,**B**) Statistical difference between the groups was analyzed by two-way ANOVA. Data are shown as mean ± SEM, *n* = 5, * *p* < 0.05; ** *p* < 0.01; *** *p* < 0.001. (**C**) The effects of *Glrx2* knockout on the protein expression levels of Trx systems in the liver from the mice treated with APAP; *n* = 4, a representative one is shown.

GSH is normally considered to play an important role in the induction of APAP hepatotoxicity [33]. Moreover, GSH functions as a component of the Grx system to reduce Grx [34]. Thus, we detected the total GSH content in the liver after the APAP challenge. APAP challenge dramatically lowered GSH content in the livers of both WT and *Glrx2^−/−^* mice at 1 h after the administration, while at 6 h post-APAP administration, GSH level in WT mice returned to physiological level and was higher than at 1 h. However, the GSH level stayed at a relatively low level in *Glrx2*-deficient mice and lower than in WT mice at 6 h post-APAP treatment (Figure 2A). This indicated that Grx2 deficiency affected intracellular redox microenvironment balance because of the crucial role of GSH in antioxidant defense [35,36]. In addition, APAP treatment caused an increase in the expression of Grx2 (Figure S1B).

The Trx system, acting as the other major thiol redox system besides the Grx system [37], was detected at both the expression and enzymatic level in the context of the impaired GSH system. There was no significant change in protein expression of the Trx system at 1 h post-APAP administration, but total TrxR activity in both WT mice and *Glrx2^−/−^* mice was largely inhibited at this time point (Figure 2B,C). Six hours after APAP treatment, TrxR activity in WT mice was restored to a higher level than 1 h. Meanwhile, TrxR activity was maintained at a lower level in APAP-treated *Glrx2^−/−^* mice (Figure 2B). Furthermore, Trx1 and Trx2 expression tended to upregulate in APAP-treated *Glrx2^−/−^* mice at 6 h (Figure 2C). Specifically, after APAP insult, Trx2 showed two bands in *Glrx2^−/−^* mice. This may indicate that APAP exposure of *Glrx2^−/−^* mice caused high oxidization of Trx2 so that the structure of part of the protein had been changed and could not be completely reduced into the initial reduced form by DTT. For example, part of Trx2 might have some type of posttranslational modification in the highly oxidative mitochondrial environment.

### 3.3. Decreased GSH/GSSG Ratio in Glrx2^−/−^ Mice after APAP Treatment Weaken Nrf2 Activation

**Figure 3:**
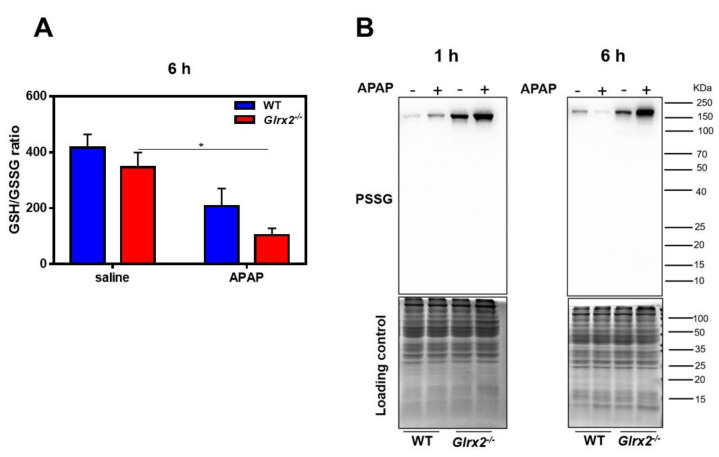
*Glrx2* knockout exacerbated proteins glutathionylation level after APAP treatment. (**A**) The ratio of reduced GSH and oxidized GSSG detected using *N*-ethylmaleimide (NEM) to fix free thiols. Statistical difference among groups was analyzed using two-way ANOVA. Data are shown as mean ± SEM, *n* = 5, * *p* < 0.05. (**B**) The change of proteins glutathionylation 1 h and 6 h after APAP treatment, which was evaluated by Western blot after separation with non-reducing SDS-PAGE. Coomassie brilliant blue staining gels as the loading control. *n* = 4, a representative one is shown.

Under normal physiological conditions, Nrf2 is inactivated by binding with Kelch-like ECH-associating protein 1 (Keap-1). When oxidative stress occurs, Nrf2 dissociates from the Nrf2-Keap1 complex and translocates to the nucleus, followed by interacting with the antioxidant response element (ARE) [38]. A previous study reported that the GSH/GSSG ratio and nuclear Trx1 controlled the dissociation/nuclear translocation of cytoplasmic Nrf2 and Nrf2/DNA interaction independently [39]; thus, we first detected the GSH/GSSG ratio in the liver tissue. In agreement with the decrease of GSH level, at 6 h after exposure to APAP GSH/GSSG, the ratio decreased significantly in *Glrx2*-deficient mice, while there was little effect on the ratio of GSH/GSSG in WT mice (Figure 3A). The result indicated that decreased GSH/GSSG ratio in *Glrx2^−/−^* mice after APAP treatment might contribute to the interruption of Nrf2 regulated compensatory response.

Decreased ratio of GSH/GSSG may lead to abnormal protein glutathionylation; therefore, we thus detected the protein glutathionylation level in livers with or without APAP exposure. After 1 h treatment with APAP, proteins glutathionylation levels in WT and *Glrx2^−/−^* mice were upregulated. Very interestingly, the level of protein glutathionylation decreased in the liver of WT mice, while it continuously increased in *Glrx2^−/−^* mice 6 h after APAP exposure (Figure 3B). These results demonstrated the deficiency of Grx2 disrupted the deglutathionylation process and exacerbated oxidative stress caused by APAP. Since protein glutathionylation participates in the biological function of many proteins [16,40], the function of these proteins thus might be affected.

### 3.4. Elevated Trx Oxidation in Glrx2^−/−^ Mice after APAP Treatment Weaken Nrf2 Activation

**Figure 4:**
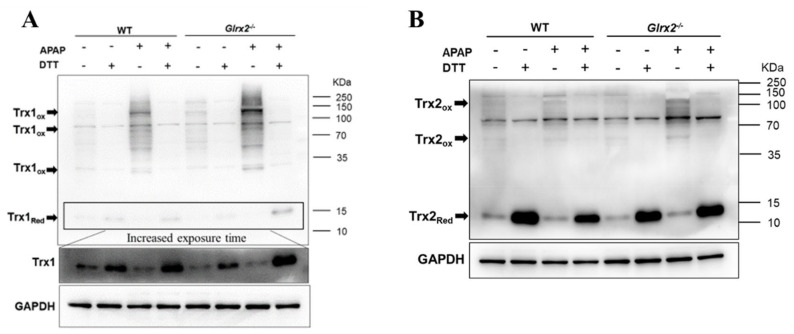
*Glrx2* knockout enhanced Trx oxidation in liver exposed to APAP for 1 h. (**A**,**B**) Trx redox state in livers was detected by Western blot in the presence or absence of 50 mM dithiothreitol (DTT). *n* = 3, a representative one is shown. GAPDH as the loading control. Arrows point to the oxidized form of Trx1/2.

The thioredoxin redox state is another indicator of the cellular redox state. Thus, we investigated the redox state of Trx using IAM to fix free thiols. Under the non-reducing condition, the free thiols in reduced Trx1/2 were alkylated by IAM to avoid oxidizing of Trx1/2 in processing samples; thus, we could find the reduced Trx1/2 in predicted molecular weight (~12 kDa). When Trx1/2 was oxidized, they would form disulfides with proteins (including Trx1/2), making them appear in the high molecular weight positions or as fuzzy bands. DTT was added to open the disulfide bond between Trx1/2 and proteins to validate that the bands in high molecular weight are specific bands of Trx1/2. In agreement with the observation of GSH content (Figure 2A) and TrxR activity (Figure 2B), both cytosolic Trx1 and mitochondrial Trx2 oxidation were enhanced in mice treated with APAP, and oxidation level in *Glrx2^−/−^* mice was higher than in WT mice after 1 h (Figure 4A,B and Figure S2) and 6 h (Figure S3) after APAP treatment. These results indicated that Grx2 deficiency caused extensive oxidative stress in both cytosol and mitochondria under exposure to APAP.

### 3.5. Glrx2 Deficiency Increased AIF Nuclear Translocation and MDA Level in Liver after APAP Administration

**Figure 5:**
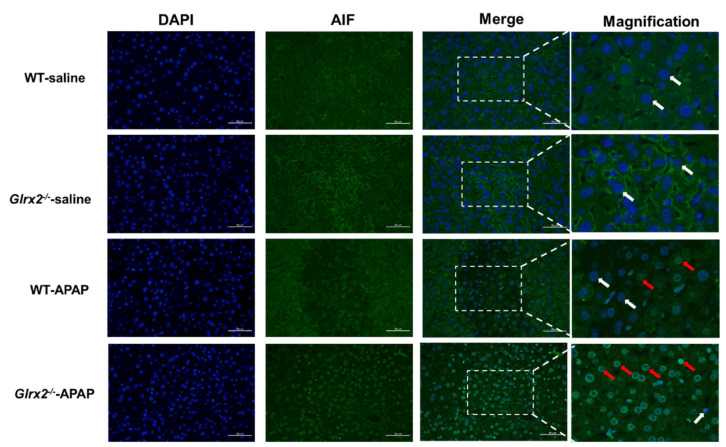
*Glrx2* knockout enhanced nuclear translocation of AIF in livers from the mice treated with APAP. The nuclear translocation of AIF in liver tissue from mice at 6 h with or without treatment with 300 mg·kg^−1^ APAP was detected by immunofluorescence. In both types of mice with saline treatment, the nuclei of DAPI appeared blue (indicated by white arrows). After APAP insult, AIF accumulated in nuclei, as shown by the merged colors (indicated by red arrows). Moreover, both the fluorescence intensity and number of nuclei with AIF increased in *Glrx2*-deficient mice-Bars, 50 μm.

Extensive oxidative stress does not only cause cellular redox compensatory response, but also cell death. In particular, Trx oxidation state is a determining factor of cell fate [41]. If the thioredoxin is changed to an oxidized state, Trx-mediated cell death pathways such as ASK1 and AIF are activated to induce cell death. Therefore, we detected the nuclear translocation of AIF with an immunofluorescence staining method (Figure 5). Under physiological conditions, AIF locates in the mitochondrial intermembrane space. Upon apoptotic stimuli, such as oxidative stress, AIF is released from mitochondria and translocated to the nucleus. DAPI indicates the cell nucleus, in the same position; AIF was found by fluorescence microscopy using AIF antibody, revealing the nuclear translocation of AIF. There were very few AIF signals in the nucleus of saline-treated mice, while a large amount of signal was found in the nucleus of APAP-treated mice, especially in *Glrx2^−/−^* mice. These results indicated that AIF translocation was activated after APAP treatment, with more AIF accumulated in the *Glrx2^−/−^* mice hepatocyte nucleus.

**Figure 6:**
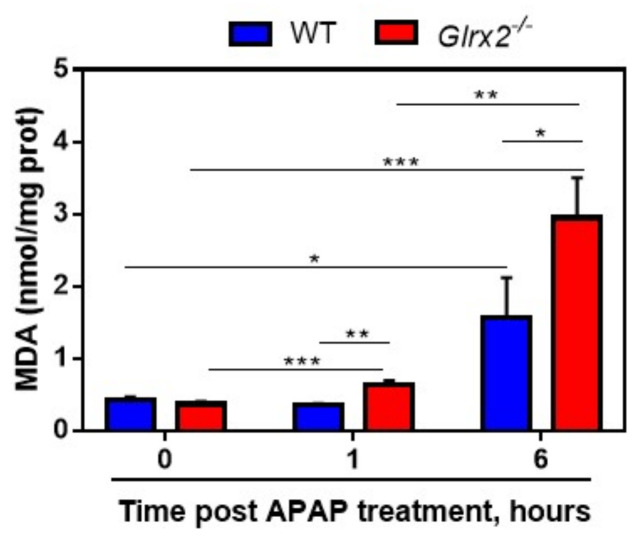
*Glrx2* knockout increased malondialdehyde (MDA) levels in APAP-treated livers. MDA level in livers from the mice exposed to 300 mg·kg^−1^ APAP was detected. Statistical difference among groups was analyzed by two-way ANOVA. Data are shown as mean ± SEM, *n* = 4, * *p* < 0.05; ** *p* < 0.01; *** *p* < 0.001.

The oxidation of GSH, the important electron donor for glutathione peroxidases (GPxs), can affect the ability of GPxs to catalyze the reduction of lipid peroxides. To prove this, we subsequently explored the level of lipid peroxide malonaldehyde (MDA) in liver. Consistent with this hypothesis, at 1 h after administration with APAP, mice with *Glrx2* deficiency displayed an elevated MDA level, higher than in APAP-treated WT mice (Figure 6). Six hours after administration, the MDA level in both WT and *Glrx2*-deficient mice were much higher than that in WT mice and in *Glrx2* knockout mice treated with APAP for 1 h (Figure 6A).

## 4. Discussion

Although the participation of GSH and mitochondria in APAP-induced hepatotoxicity had been well studied, the role of Grx2, a mitochondrial GSH system component, had not yet been reported. In this study, we found that *Glrx2* deficiency aggravated APAP-caused liver damage, indicating the critical role of Grx2 in protecting the liver from APAP-induced oxidative damage. To further explore the mechanism, higher levels of proteins glutathionylation and Trx oxidation were found in *Glrx2*^−/−^ mice demonstrating that Grx2 exerted its potential protective role by regulating Trx and GSH systems activity. Based on this and other related studies [29,42], a mechanism about why *Glrx2* KO augmented hepatotoxicity in mice after the APAP challenge was proposed. Initially, APAP metabolite, *N*-acetyl-*p*-benzo-quinonimine (NAPQI), inhibited GSH level and TrxR activity, and lower GSH level resulted in a decreased GSH/GSSG ratio in mice, which caused the activation of Nrf2-mediated redox compensatory response to enhance the expression of TrxR1, GCLC, and GCLM, which are responsible for the synthesis of GSH. This feedback response resulted in antioxidant capacity recovery in the cytosol and a complete Grx and Trx system in the mitochondria, thus actively removing excessive ROS and maintaining cellular redox balance. Under the Grx2 KO condition, the redox homeostasis in mitochondria was interrupted, and the total mitochondrial antioxidant capacity was affected. Upon the challenge with APAP, the interruption of mitochondrial redox homeostasis may thus contribute to the elevation of the cellular ROS level and result in extensive cellular oxidation. Thus, it can be indicated that both cytosolic and mitochondrial Trxs became more oxidized (Figure 4). The excessive ROS production may also block the activation of Nrf2 and delay the recovery of TrxR activity and GSH level. Furthermore, the disruption of the mitochondrial Grx2 pathway switches the cell fate towards the cell death process. In our study, we found that *Glrx2* knockout enhanced the nuclear translocation of AIF. This indicated that AIF-mediated cell death, in which activity is regulated by the thioredoxin redox state, was activated following the oxidation of Trxs.

Our results suggest that *Glrx2* deficiency sensitized mice to APAP intervention by disrupting the compensatory regulation of redox antioxidant systems. These results were consistent with the findings from other research groups that *Glrx2* deficiency exacerbated oxidative stress in mouse lens epithelial cells [24]. In detail, when cells were treated with hydrogen peroxide (H_2_O_2_) for 6 h, the GSH content in *Glrx2* deleted mouse lens epithelial cells was significantly lower than that in WT cells, which displayed a similar change to that in the liver of APAP-treated mice in this study (Figure 2A). In addition to GSH content, enhanced protein glutathionylation was found in *Glrx2^−/−^* mice. Notably, *Glrx2* knockout increased glutathionylation levels in the kidney (data not shown) and liver without APAP treatment, according to our results (Figure 3B) and other studies [25]. However, it had no effect on H_2_O_2_-free lens epithelial cells. Different regulation patterns after *Glrx2* deletion in various tissues may account for this difference. Moreover, the study of Mailloux and colleagues showed that Grx2 deficiency did not induce a compensatory overexpression of Grx1 in the liver [25]. This may indicate that the role of Grx2 in the regulation of liver redox balance is unique. Mitochondrial protein complex I was found to be modified by GSH [17], and the loss of complex I activity resulting from its glutathionylation was considered to be the main cause of increased sensitivity to stress in *Glrx2* deficiency cells [24] or tissues [25]. This is in agreement with results that displayed morphological and functional alteration in Glrx2-deficient liver mitochondria [26].

In parallel to the GSH system, another disulfide reductase system, the Trx system, composed of NADPH, TrxR, and Trx, functions as the backup of the GSH system to maintain the intracellular redox balance. For instance, Grx2 could catalyze the reduction of oxidized Trx1 and Trx2 in cells exposed to TrxR inhibitors and thus enhance cell viability [43]. Since this crosstalk between these two systems, the greater challenge was raised for the discovery of anticancer drugs targeting TrxR, but therapy strategy for certain kinds of cancers with loss of expression of enzymes required for GSH homeostasis [44] was also put forward. Unfortunately, the majority of reports related to *Glrx2* deficiency focused on the effects on the GSH system, neglecting the influence on the Trx system. Here, we also detected the Trx system activity implied by TrxR activity and Trx redox state and found increased Trx1/2 oxidation in APAP-treated *Glrx2^−/−^* mice. This was in agreement with our previous study [29] that revealed the correlation between Trx oxidation and liver damage. Loss of Grx2 reduction activity to Trx, resulting from *Glrx2* deficiency, may support proof for this enhancement in Trx oxidation. Further results documented that the nuclear translocation of AIF was induced by APAP treatment, and more signals were found in *Glrx2^−/−^* mice (Figure 5). As a Trx regulated protein, AIF combines with reduced Trx under the non-stressed condition, but dissociates from the complex when Trx is oxidized, then translocates from the mitochondria to the nucleus to induce apoptosis [42].

## 5. Conclusions

*Glrx2* deletion augmented effects on the cellular redox microenvironment indicated by decreased total GSH content and TrxR activity, thus exacerbating APAP hepatotoxicity. The Grx2 deletion caused the interruption of redox homeostasis and decreased the antioxidant capacity in mitochondria. Upon exposure to APAP, this predisposition of Grx2 deletion thus enhanced oxidative stress and delayed the activation of Nrf2-mediated feedback redox response. This decreased the GSH/GSSG ratio and increased Trx oxidation, triggering the AIF-mediated cell death pathway. The limitation of this study is that the exact mechanism of the role of Grx2 in APAP-induced liver damage has been illuminated; however, the strategy via enhancing Trx and GSH systems mediated activities to detoxify APAP needs further investigation. In addition, the contribution and crossing roles of Grx2, Grx1 and Trx1, Trx2 in the regulation of AIF translocation and the related apoptosis pathway are also very intriguing and need further clarification.

## Data Availability

All of the data is contained within the article and the Supplementary Materials.

## Supplementary Materials

The following are available online at https://www.mdpi.com/article/10.3390/antiox11091643/s1, Figure S1: *Glrx2* knockout mice identification; Figure S2: Quantification of Trx1/2 redox state; Figure S3: *Glrx2* knockout enhanced Trx oxidation in liver from the mice exposed to APAP for 6 h.
