# Supplementary material for: Mitochondrial Glrx2 Knockout Augments Acetaminophen-Induced Hepatotoxicity in Mice
Source: Antioxidants (Basel). 2022 Aug 24;11(9):1643. doi: 10.3390/antiox11091643 (PMC9495392; doi:10.3390/antiox11091643)
Supplement: Supplementary file 1 [file antioxidants-11-01643-s001.zip › antioxidants-1832535-supplementary.pdf]

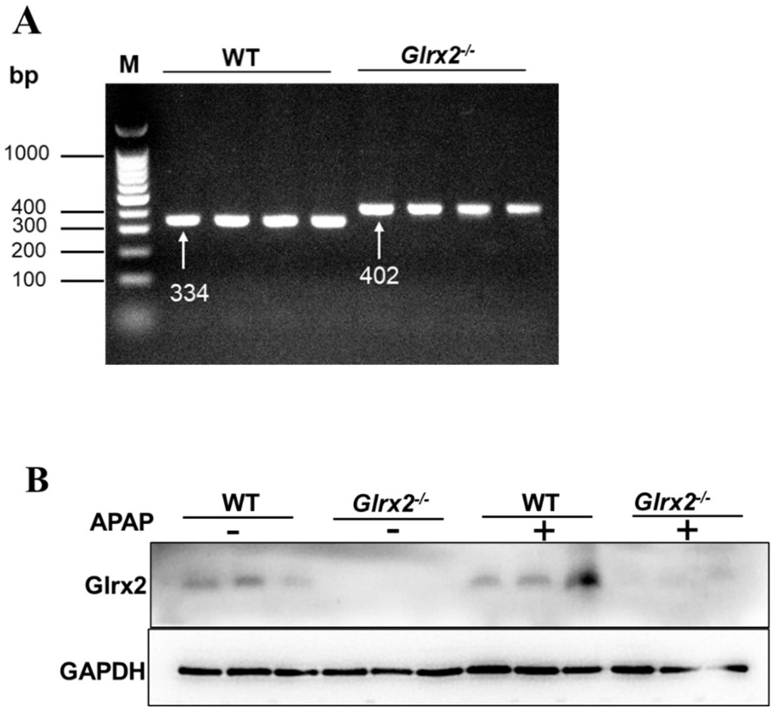

Figure S1 Identification of *Glrx2* knockout mice and the effect of APAP on the expression of Grx2 in mouse liver. (A) genotyping, n=4. (B) Grx2 protein expression in liver from mice with or without APAP treatment for 24 h, n=3.

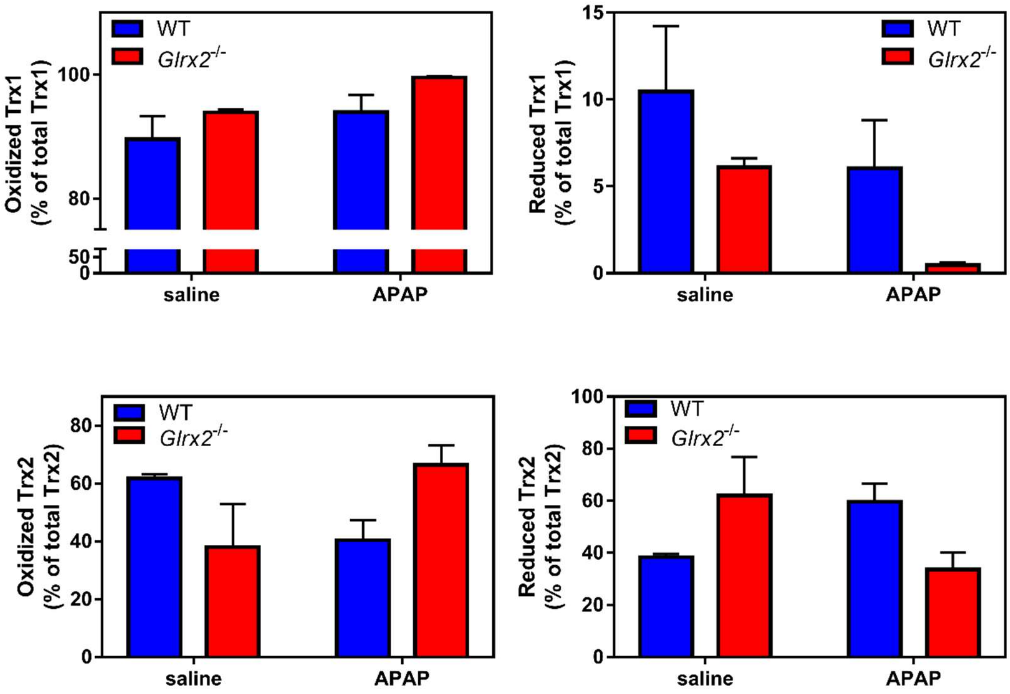

Figure S2 Quantification of Trx1/2 redox state. The amount of oxidized and reduced Trx1/2 in mice liver was quantified by Image J (*n*=3).

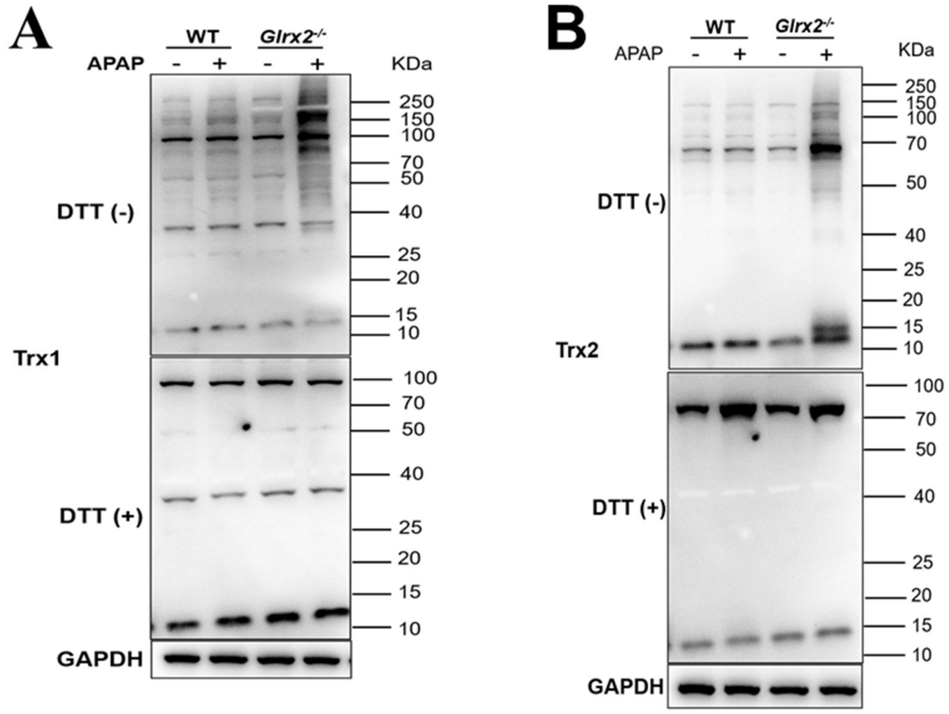

Figure S3 *Glrx2* knockout enhanced Trxs oxidation in liver from the mice exposed to APAP for 6 h. *n*=3, a representative one is shown. GAPDH as the loading control.
